# Smoking as a risk factor for colorectal neoplasms in young individuals? A systematic meta-analysis

**DOI:** 10.1007/s00384-023-04405-w

**Published:** 2023-05-06

**Authors:** Qiang Li, Jutta Weitz, Chao Li, Josefine Schardey, Lena Weiss, Ulrich Wirth, Petra Zimmermann, Alexandr V. Bazhin, Jens Werner, Florian Kühn

**Affiliations:** 1 Department of General, Visceral, and Transplant Surgery, Ludwig-Maximilians-University Munich, 81377 Munich, Germany; 2 Department of Medicine III, University Hospital, Ludwig-Maximilians-University Munich, 81377 Munich, Germany; 3 German Cancer Consortium (DKTK), Partner Site Munich, 81377 Munich, Germany; 4 Bavarian Cancer Research Center (BZKF), Partner Site Munich, 81377 Munich, Germany

**Keywords:** Early-onset colorectal neoplasms, Risk factor, Smoking

## Abstract

**Background and aims:**

Early-onset colorectal neoplasms (EoCRN) include both benign and malign colorectal tumors, which occur before the age of 50. The incidence of EoCRN is rising worldwide. Tobacco smoking has previously been proven to be related to the development of various tumor types. However, its relationship with EoCRN is not clearly defined. Hence, we carried out a systematic review and a meta-analysis to evaluate the relationship between smoking status and the risk of EoCRN.

**Methods:**

A systematic search of PubMed, EMBASE, and Web of Science up to September 7, 2022, was performed for studies that evaluated the association of smoking status with EoCRN. The quality of the case–control study was evaluated with the Newcastle‒Ottawa Scale. The quality of the cross-sectional studies was evaluated with the American Health Care Research and Quality checklist. Fixed-effects models were used to pool odds ratios (ORs) to evaluate the relationship between the risk of developing EoCRN and smoking status. The meta-analyses were performed with Review Manager version 5.4, and funnel plots and publication bias tests were produced by STATA software.

**Results:**

A total of six studies were included in this meta-analysis. After pooling the results of these six studies, we found that current smokers carry a relatively high risk of developing EoCRN (OR, 1.33; 95% confidence interval [CI], 1.17–1.52) compared to never-smokers. Ex-smokers were not at a significantly increased risk for developing EoCRN (OR, 1.00; 95% CI, 0.86–1.18).

**Discussion:**

Smoking behavior is significantly associated with an increased risk for developing EoCRN and might be one of the reasons for the increasing incidence. Ex-smokers who quit are not at significant risk of developing EoCRN.

**Supplementary Information:**

The online version contains supplementary material available at 10.1007/s00384-023-04405-w.

## Introduction

Early-onset colorectal neoplasms (EoCRN) are a group of abnormal growths that form in the large intestine, including both benign and malignant tumors, and occur before the age of 50 [1]. Among these, colorectal adenomas are the most common benign tumors, while colorectal cancer (CRC) is the most prevalent malignant tumor [1]. The incidence and mortality rates of CRC have generally decreased [2, 3], in part due to increased screening among average-risk adults beginning at age 50 [4, 5], as well as positive changes in certain lifestyle risk factors [6]. Early-onset colorectal cancer (EoCRC) refers to the development of CRC before the age of 50 [7]. It is an emerging global health concern and tends to have a worse prognosis compared to CRC which develops later in life [2, 3]. While the incidence of CRC has been declining in recent years, there has been a significant increase in the incidence of EoCRC in the USA, China, Australia, Brazil, the UK, and Japan [8-10]. Timely detection and treatment of EoCRN are crucial, as adenomas have the potential to develop into adenocarcinomas over time, which can result in a more severe prognosis [11].

While the exact causes of EoCRN are not fully understood, several risk factors have been identified, including smoking [12, 13]. Smoking is a known risk factor for several types of cancer [14-16], but its association with EoCRN remains unclear, and previous meta-analyses have reported conflicting results [7, 17]. Recently, additional studies on the risk of smoking and EoCRN have been published [12, 18]. Therefore, an updated meta-analysis is necessary to synthesize the current evidence and provide more reliable insights into the association between smoking and the risk of developing EoCRN. The findings of this updated meta-analysis will be valuable in guiding public health efforts to prevent EoCRN, particularly in populations with high rates of smoking.

## Methods and materials

We carried out this systematic meta-analysis on the basis of the PRISMA guidelines [19]. The program for conducting the systematic review and meta-analysis was registered in PROSPERO with the registration number (CRD42022367875).

### Literature search

We have systematically searched PubMed, EMBASE (OVID), and Web of Science as of September 7, 2022, with a search strategy based on “Colorectal Neoplasm”, “Early-onset”, “Risk”, and “Smoking”. To include as many relevant studies as possible, studies related to smoking and the risk of EoCRN, which were referred to other meta-analyses [7, 17], were also included. No publication status or publication date restrictions were imposed, but we limited the language of the study to English. Further information on the search strategy is presented in Supplementary Appendix 1.

### Study eligibility

In this systematic review, the study population was less than 50 years old at the time of study entry, and all those studied were younger than 55 years old at the initial diagnosis of CRN. The exposed group was defined as the smoking population and the former smoking population, and the non-exposed group was defined as the non-smoking population. Original studies reporting multivariate ORs, RRs, or hazard ratios (HRs) values for the association between smoking status (current smoking, former smoking, and non-smoking) and the risk of developing EoCRN were deemed eligible for inclusion. All cases included in the study were diagnosed through histological or pathological examination by colonoscopy. This analysis included studies that excluded individuals with a family history of CRC, as well as studies that calculated ORs, RRs, or HRs for the association between smoking status and risk of EoCRN using multivariable logistic regression adjusted for CRC family history to eliminate the potential interference. The study type included was observational, including cross-sectional and case–control studies.

Exclusion criteria were as follows: (1) conference abstracts, reviews, comments, case reports, or letters were excluded; (2) passive smoking; (3) duplicate literature; (4) studies without complete information; and (5) non-English studies.

### Data extraction and quality assessment

Two authors (Qiang Li and Chao Li) independently extracted the data from eligible studies and then communicated about the differences to obtain the final data (Table 1). The data were extracted from eligible studies including author, publication year, study type, country, sex, recruitment age, age at diagnosis of EoCRN, sample size, tumor type, tumor sites, smoking status, outcome (ORs/RRs/HRs, 95% CI), covariates, case confirmation, matching controls, and follow-up time. Case–control studies’ quality was measured with the Newcastle‒Ottawa Scale (NOS) [20]. Cross-sectional studies were evaluated for quality with the American Health Care Research and Quality (AHRQ) methods checklist [21].

**Table 1:** Characteristics of the included studies

**First author, year**	**Population selection**	**Country**	**Sex**	**Recruitment age**	**Age (time) at diagnosis of EOCRC**
Shen et al. (2021) [12]	Population-based	China	M/W	<50	<54
Low et al. (2020) [15]	Veteran-based	The US	M/W	18-49	18-49 (1999-2014)
Agazzi et al. (2021) [18]	Hospital-based	Italy	M/W	18-49	18-49
Lee et al. (2016) [23]	Hospital-based		M/W	-49	<51
Koo et al. (2017) [13]	Population-based	Korea	M/W	40-49	40-49
Kawk et al. (2015) [22]	Employees-based population	Korea	M/W	20-39	<41

**N**_**total**_**(n**_**casea**_**)**	**Tumor type**	**Case confirmation**	**Matching of controls**	**Result ** **______________________** **Smoking status OR(95%C I)**	**Covariate**
17293 (124)	neoplasm	Pathology reports of colonoscopy	Age, sex	Current-smoking 0.92(0.27-3.12)	Sex, BMI, Family history of CRC
				Ex-smoking 1.27(0.75-2.15)	
68067 (651)	cancer	colonscopy	Veterans free of CRC	Current-smoking. 1.10(0.89-1.35)	Age,Sex , BMI, Aspirin use
				Ex-smoking. 0.82(0.60-1.12)	
1778 (223)	adenoma and adenocarcinoma	colonscopy	Age, sex	Current-smoking 1.10(0.65-1.86)	Sex, Age, Family history of CRC, Alarm symptoms, GI-symptoms, IBD
				Ex-smoking 0.89(0.50-1.58)	
1776 (253)	adenoma	Colonscopy histologically	Age,sex	Current-smoking 1.60(1.07-2.41)	Age, Sex, BMI, ALC, HTN, MS, TG, HDL, Waist circumference, Diabetes mellitus, High hsCRP , LDL,
				Ex-smoking 1.23(0.79-1.93)	Total cholesterol

*BMI* body mass index, *ALC* alcohol consumption, *HTN* hypertension, *HDL* high-density lipoprotein, *LDL* low-density lipoprotein, *TG* triglyceride, *GI* gastrointestinal, *IBD* inflammatory bowel disease, *hsCRP* high-sensitivity C-reactive protein, *FPG* fasting plasma glucose

### Data synthesis

Among the six studies that were included, two investigated CRN [12, 13], two studied colorectal adenoma [22, 23], one investigated both colorectal adenoma and adenocarcinoma [18], and one focused on CRC [24]. The included studies provided only OR values and 95% CI for the relationship between smoking status and the risk of EoCRN, and no eligible studies provided corresponding RR and HR values, which is why only effect sizes for OR values were combined. The multivariate OR values extracted from each study were transformed into the natural logarithm, and their standard errors were calculated based on ﻿the logarithmic numbers and their corresponding 95% CIs. The OR values were pooled with both the fixed effects model and the random effects model. The fixed effects model was finally used for further analysis because of the low ﻿heterogeneity in the included studies [25].

﻿The study group was divided into current smokers, former smokers, and non-smokers based on the description of the individuals. Current smokers were defined as subjects who smoked a minimum of one cigarette a day, regardless of the type of cigarette. Former smokers were defined as study cases who did not smoke for at least 1 year prior to inclusion in the study but did consume at least one cigarette per day in the past, regardless of the type of cigarette. Never-smokers mean that the subject has never actively smoked any cigarettes. The possibility of publication bias was evaluated by inspecting funnel plots [26]. The meta-analysis was carried out with Review Manager version 5.4. All *P* values were bilateral, and the significant level has been fixed at 0.05.

## Results

### Search result

**Fig. 1:**
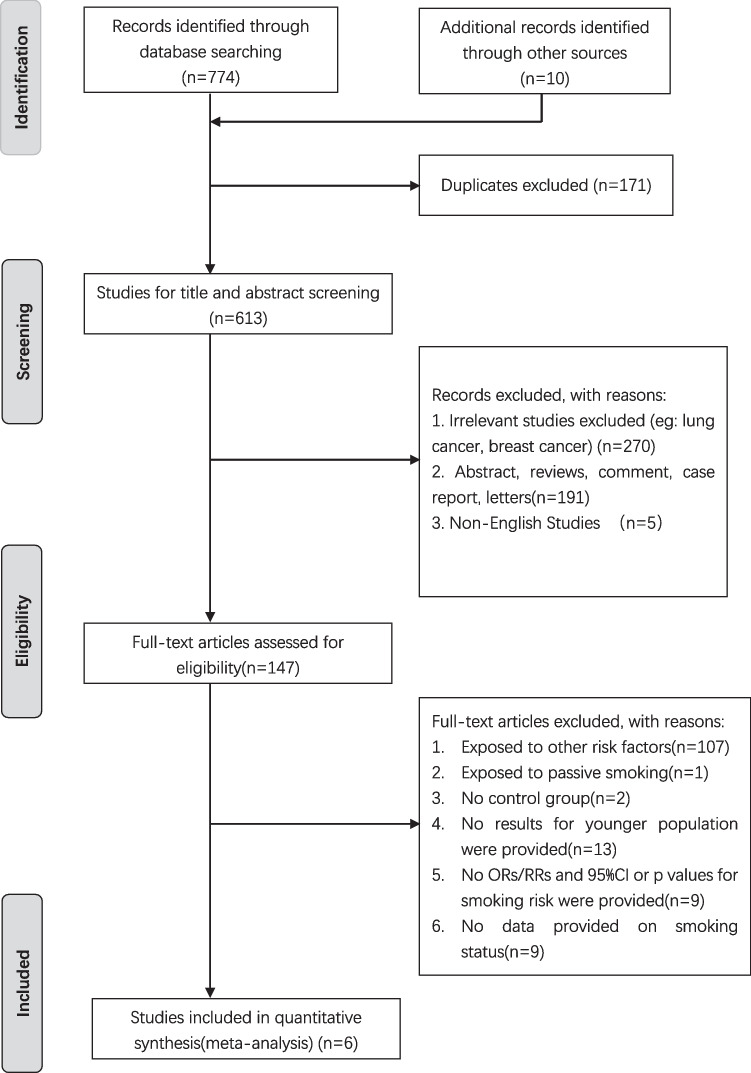
﻿Flow chart of selection procedure of studies assessing the relationship of current smoking and ex-smoking with the risk of developing early-onset colorectal neoplasms

A total of 613 publications were included in the study via database search and references from other meta-analyses. After a cursory screening of study titles and abstracts, 466 articles were excluded depending on the inclusion and exclusion criteria. After a thoroughly detailed review of the 147 remaining articles, 141 articles were excluded for insufficient study data. Finally, 6 eligible studies [12, 13, 18, 22-24] were included. The selection process is detailed in Fig. 1.

### Patient characteristics in eligible studies

As shown in Table 1, a total of 6 articles with 95,406 patients from 4 different countries were included in this study. The sample size of the included studies varied between *n* = 1776 and 68,067. Patients enrolled in the study were younger than 50 years of age. The follow-up time ranged from 12 to 48 months.

### Risk of bias assessment

The quality of the included articles was measured with NOS [20] based on 3 items: selection, comparability, and outcome. The scores for case–control studies ranged from 0 to 9. A high score represents the high quality of the included study. NOS scores of 0–3, 4–6, and ≥ 7 were defined as representing low, medium, and high quality, respectively. The cross-sectional studies were assessed in terms of their quality with the AHRQ methodology checklist [21]. The checking scale consists of 11 events, with the risk of bias represented by a score of 1 representing “yes” and 0 indicating “unclear” or “no” risk. Following the recording of the overall score, the articles were categorized into 3 levels: “Low” (0–3 scores), “Medium” (4–7 scores), and “High” (8–11 scores). The risk of bias in the 4 case–control studies and the 2 cross-sectional ones is shown in Table 2.

**Table 2:** Risk of bias of case–control study and risk of bias of the cross-sectional study

Author, year^a^			Selection			Comparability			Outcome			Total score
Shen et al. (2021) [12]			★★★★			★★			★★			8
Low et al. (2020) [15]			★★★★			★★			★★			8
Agazzi et al. (2021) [18]			★★★★			★			★★			7
Lee et al. (2016) [23]			★★★★			★			★★			7
Author, year^b^	1	2	3	4	5	6	7	8	9	10	11	Scores
Koo et al. (2017) [13]	N	N	N	N	Y	N	Y	Y	Y	Y	Y	6
Kwak et al. (2015) [22]	N	N	N	Y	Y	Y	Y	Y	Y	Y	Y	8

The quality of the included articles was measured with the NOS for case–control studies (a) and with the AHRQ methodology checklist for cross-sectional studies (b). In (a), each star represents a point. In (b), each listed event with the risk of bias is represented by a Yes (Y) or No (N)

### Smoking and the risk of EoCRN

**Fig. 2:**
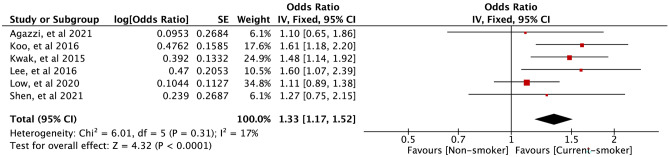
﻿Association of smoking (current smokers vs non-smokers) with developing EoCRN risk. The result from the fixed effects model with a sample size of 95406. EoCRN, early-onset colorectal neoplasms; CI, confidence interval; OR, odds ratio

**Fig. 3:**
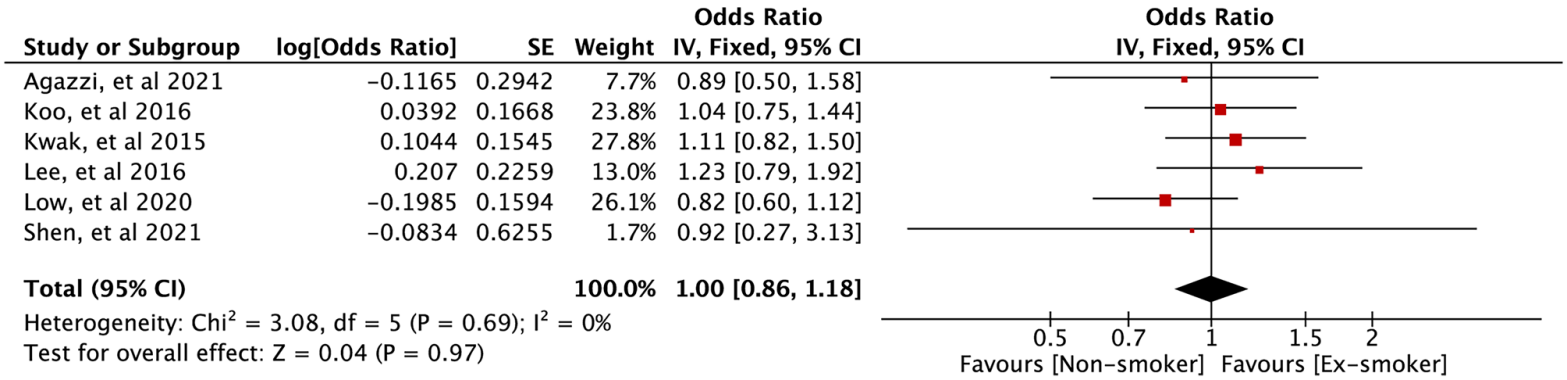
﻿Association of ex-smoking (ex-smokers vs non-smokers) with developing EoCRN risk. The result from the fixed effects model with a sample size of 95406. EoCRN, early-onset colorectal neoplasms; CI, confidence interval; OR, odds ratio

Six studies provided data on smokers (current and non) and EoCRN risk. The forest plot (Fig. 2) shows a positive association between current smokers and EoCRN compared to non-smokers (OR = 1.33, 95% CI = 1.17–1.52, *P* < 0.0001). In addition, these 6 studies [12, 13, 18, 22-24] reported data on smokers (ex and non) and the risk of EoCRN. The forest plot (Fig. 3) shows that there is no significant correlation observed between ex-smokers and EoCRN in comparison to non-smokers (OR = 1.00, 95% CI = 0.86–1.18, *P* = 0.97).

**Fig. 4:**
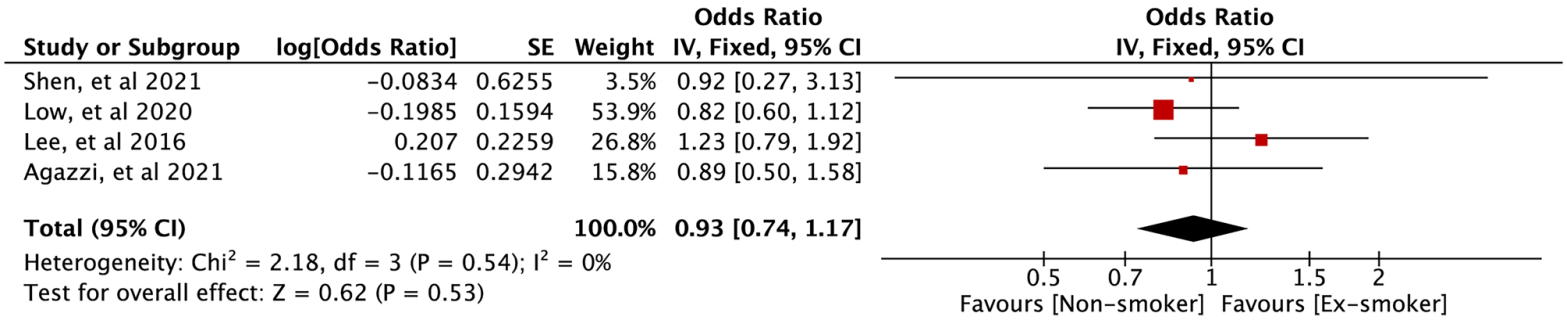
Association of smoking (current smokers vs non-smokers) with developing EoCRN risk in case–control studies. The result from the fixed effects model with a sample size of 88914. EoCRN, early-onset colorectal neoplasms; CI, confidence interval; OR, odds ratio

**Fig. 5:**
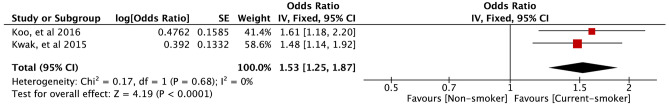
Association of smoking (current smokers vs non-smokers) with developing EoCRN risk in cross-section studies. The result from the fixed effects model with a sample size of 6492. EoCRN, early-onset colorectal neoplasms; CI, confidence interval; OR, odds ratio

Four case–control studies [13, 22-24] compared current smokers against non-smokers, and two cross-sectional studies [12, 18] compared current smokers against non-smokers. Figures 4 and 5 show a meta-analysis of the relationship between current smokers and the risk of EoCRN compared to the relationship between non-smokers and the risk of EoCRN in case–control studies and cross-sectional studies, respectively. The association between current smokers and EoCRN compared to non-smokers is positive in case–control studies (OR = 1.20, 95% CI = 1.01–1.43, *P* = 0.04, see Fig. 4), as well as in cross-sectional studies (OR = 1.53, 95% CI = 1.25–1.87, *P* < 0.0001, see Fig. 5). Figures S1 and Figure S2 show the meta-analysis of the findings on the relationship between ex-smokers and the risk of EoCRN compared to non-smokers in case–control studies and cross-sectional studies, respectively. There is no significantly correlated relationship between ex-smokers and EoCRN, compared with non-smokers in neither the case–control study OR = 0.93, 95% CI = 0.74–1.17, *P* = 0.53, see Fig. S1) nor in the cross-sectional study (OR = 1.08, 95% CI = 0.86–1.35, *P* = 0.51, see Fig. S2).

Four studies were conducted in Asia [12, 18, 23, 24], and two studies [13, 22] were conducted in Europe and the USA. Figures S3 and S4 show the risk assessment for EoCRN of current smokers compared to non-smokers in different regions. In the studies conducted in Asia, a positive association between current smokers and EoCRN compared to non-smokers is found (OR = 1.51, 95% CI = 1.28–1.79, *P* < 0.00001, see Fig. S3). In the studies conducted in America and Europe, there is no significant correlation between current smokers and EoCRC compared to non-smokers (OR = 0.84, 95% CI = 0.63–1.10, *P* = 0.20, see Fig. S4). Figures S5 and S6 show correlations of the risk of EoCRN in current smokers compared to non-smokers in different regions. There is no significant correlation between ex-smokers and EoCRN compared to non-smokers, neither in Asia (OR = 1.00, 95% CI = 0.91–1.34, *P* = 0.34, see Fig. S5) nor in America and Europe (OR = 1.11, 95% CI = 0.90–1.36, *P* = 0.32, Fig. S6).

### Publication bias

**Fig. 6:**
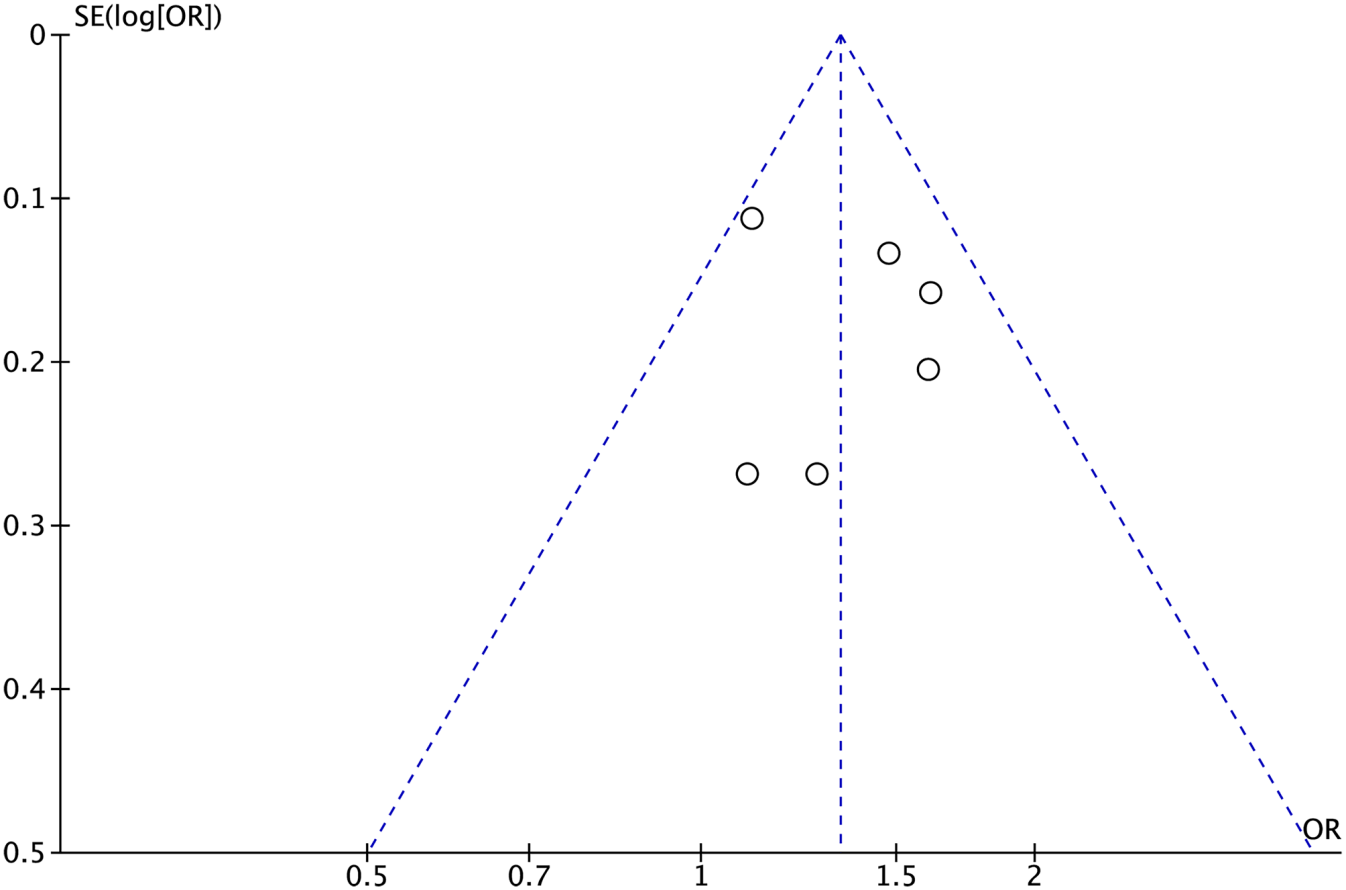
Funnel plot for the evaluation of potential publication bias in the impact of current smoking and the risk of early-onset colorectal neoplasms

Visual inspection indicated that the funnel plot for the risk of EoCRN in current smoker patients was symmetrical. During the formal statistical tests, including Egger’s test (*P* = 0.726) and Begg’s test (*P* = 0.851), there was no publication bias. The funnel plot is shown in Fig. 6.

## Discussion

### Main findings

The analysis of six studies showed a statistically significant relationship between cigarette smoking and the risk of EoCRN, but no significantly higher incidence of EoCRN in patients who had quit smoking compared to non-smokers.

A subgroup analysis was conducted by study type, including case–control and cross-sectional studies. In both types of studies, current smoking was found to be significantly linked with an increased risk of EoCRN, while the incidence of EoCRN did not show a significant increase in former smokers when compared to non-smokers. Another subgroup analysis was performed based on the geographical region (Asia, Europe, and America) of the studies. The results showed that the association between current smoking and the risk of EoCRN was statistically significant in Asia, but not in Europe and America, compared to non-smokers. Furthermore, there was no significant increase in the risk of EoCRN among former smokers compared to non-smokers in both geographic subgroups. The differences in smoking habits, frequency, and duration among populations in Europe and the USA, as well as variations in tobacco production standards between regions, may have contributed to these inconsistent results [27]. Moreover, given the limited number of studies conducted in Europe and America, the findings from the subgroup analysis may be considered false negatives. Therefore, more high-quality studies are needed to analyze the relationship between smoking habits, age of smoking onset, duration and frequency of smoking, and the risk of EoCRN in different regions.

The studies included in the analysis evaluated potential confounding factors such as alcohol consumption, family history, and body mass index while investigating the association between smoking and EoCRN. The pooled results of this meta-analysis indicate that the association between current smoking and EoCRN remained positive even after adjusting for these confounding factors. While the study did not assess smoking-related increases in EoCRN mortality due to the lack of reported mortality rates for EoCRN, a large-scale investigation has linked cigarette smoking with a higher mortality rate for CRC [28]. Additionally, various tobacco control measures have been associated with gradual and long-lasting reductions in cancer mortality [29]. In summary, smoking represents a significant risk factor for the development of CRN in younger individuals.

### Molecular data support an association between smoking and CRC (Table 3)

**Table 3:** Smoking-related molecular mechanism and CRC risk

**Related molecular**	**Sample type**	**Main methods**	**Related mechanism**	**Ref**
**p53 gene**	Human colorectal tumor tissues	Direct sequencing; immunohistochemistry	Smoking is associated with a higher frequency of p53 deletion mutation	[31]
**﻿DNA microsatellite markers; CpG island; BRAF**	Subjects’ tumor and tumor tissue	PCR; immunohistochemical stain	Smoking was associated with epigenetic modification (the MSI-high, CIMP-positive, and BRAF mutation)	[32]
**﻿APC gene; hMLH1**	Tumor tissue of CRC patients	PCR; hMLH1 immunohistochemistry	The association between smoking and CRC risk depends on depend on molecular characteristics of the tumor (APC mutation; lack of hMLH1 expression)	[33]
**﻿Leptin gene**	Subjects’ peripheral blood samples	Genotyping assay	Smoking strengthened the association between polymorphisms in LEPR and risk of CRC	[34]
**﻿APC gene**	Subjects’ tumor tissue and mucosa adjacent to tumors	﻿Illumina infinium human methylation 450 bead-chip microarrays	Smoking is associated with ﻿hypermethylation of the key tumor suppressor gene APC	[35]
**rs1957636 at 14q22.3; rs4813802 at 20p12.3 in man; rs6687758 at 1q41, rs174537 at 11q12.2, rs4813802 in woman**	Subjects’ blood samples	Agenabio mass-array iPLEX® gold assay	Smoking behaviors modify the association between susceptibility SNPs and CRC risk	[36]
**﻿miR-21; Claudin-1; E-Cadherin**	Caco-2 cell line	MTS assay; flow cytometry; RT-PCR; permeability assay; invasiveness assay	Cigarette smoke extract increasing miR-21, Claudin-1, and E-Cadherin and enhancing the aggressiveness of cancer cells	[39]
**﻿miR‐200c**	Human Caco2, LS174T, HT‐29, and SW620 CRC cells lines	Transfection of miRNA mimics; qRT‐PCR; Western blot; cell proliferation assays; wound healing assay; transwell assay	Nicotine promotes growth and metastasis in CRC by downregulating miR‐200c	[40]
**IL-22**	Peripheral blood sample of CRC patients; CRC tissues and attached normal gut tissues; ﻿HT29 and LoVo cell lines	﻿Flow cytometry and live cell sorting; Luminex and ELISA	﻿﻿Chronic smokers may have higher risk for CRC and worse prognosis due to dysregulated IL-22 production	[41]

*PCR* polymerase chain reaction, *MSI* ﻿microsatellite instability, *CIMP* *CpG* island methylator phenotype, *qRT‐PCR* quantitative reverse‐transcription polymerase chain reaction, *SNPs* single-nucleotide polymorphisms

Tobacco smoking has consistently been the predominant exposure factor impacting gene-environment interactions in cancer [30]. Recently, many studies have suggested that some key gene mutations related to a high CRC risk are modified by smoking behavior [31-36]. The p53 and BRAF (v-raf murine viral oncogene homolog B1) gene mutations have been commonly encountered in CRC and are affected by exogenous etiological factors [31, 32]. Smoking has a significant statistical association with p53 and BRAF mutations [31, 32]. The adenomatous polyposis coli (APC) gene has been considered one of the key driver genes, like p53 and BRAF [37]. APC mutations have been found to be correlated with smoking and CRC risk in a statistically significant way [33]. Furthermore, the percentage of CRC patients in active smokers with APC 1A promoter hypermethylation was significantly higher than in former smokers and never-smokers [35]. The duration of smoking also has a significant statistical association with the hypermethylation of the APC 1A promoter [35]. The APC pathway was reported to be an independent pathway from ﻿microsatellite instability (MSI), which was identified as the main type of mismatch repair loss in tumors [38].

A lack of human mutL homolog 1 (hMLH1) was reported in approximately 90% of microsatellite-unstable tumors [33]. In the smoking-associated pathway of CRC, there was a statistically significant link between smoking and hMLH1 status [33]. ﻿The length of time smoked and the average daily amount of smoking were also significantly associated with ﻿CpG islands (CGIs) methylator phenotype-positive CRC subtypes [32]. Moreover, smoking behaviors modified the association between susceptibility to single-nucleotide polymorphisms and the risk of CRC, even though the related genotypes are different in male and female individuals [36]. Smoking also amplifies the association between polymorphisms in the leptin receptor and CRC risk [34].

In addition to altered genetic phenotypes, a variety of protein and ﻿microRNAs (miRNAs)-related molecular mechanisms have been shown to be connected to smoking in CRC [39, 40]. Cigarette smoke extract can promote the aggressive ability of CRC by increasing not only Claudin-1 and E-cadherin but also microRNA-21 (miR-21) in vitro [39]. Nicotine downregulated micro ribonucleic acid-200c (miR-200c) to promote growth and metastasis of CRC in various human CRC cell lines [40]. It has been reported that the cytokine interleukin-22 (IL-22) could not only protect the intestinal epithelium integrity but was also related to the occurrence and development of CRC by various pathways [41]. ﻿Aryl hydrocarbon receptor (AHR), which is sensitive to polycyclic aromatic hydrocarbons controls interleukin 22 production by T helper 17 cells (Th17) and T helper cells type 22 (Th22) [42, 43]. In smoking CRC patients, there were higher serum levels of IL-22 and increased IL-22 production in normal gut tissues than in non-smoking CRC patients [41]. In conclusion, an increasing number of molecular mechanisms provide causal explanations for the association between smoking and CRC.

## Strength and limitation

The results from the latest published analyses on EoCRN risk showed a controversial role of smoking in EoCRN risk [7, 17]; this study re-evaluated the role of smoking in relation to EoCRN risk. However, the present study has also some limitations. First, there are only a limited number of studies that have investigated various types of EoCRN, making it difficult to conduct subgroup analyses on different stages of EoCRN, such as colorectal adenoma and CRC. As a result, it is challenging to assess the specific risk of smoking for different stages of EoCRN. Second, many of the included studies were retrospective clinical trials, and important information may be missing. Third, many of the included studies were of relatively small sample size and had a short duration of follow-up. Fourth, the patient populations included did differ. Some studies were based on community-based populations, some on veterans, and some on colposcopy-screened populations. These factors may affect the robustness of the results. Therefore, further research is necessary to assess the association between various types of EoCRN and the risk of smoking. Moreover, more studies are needed to investigate the effects of smoking duration, frequency, and long-term outcomes on the development EoCRN.

## Conclusion

The study showed that current smoking had a statistically significant impact on the risk of developing EoCRN. Along with other lifestyle factors, this may be one reason for the rising incidence of EoCRN. Ex-smokers did not have a statistically significant risk for developing EoCRN compared to non-smokers, which underscores the need for effective communication about the benefits of a tobacco-free lifestyle.

### Supplementary Information

Below is the link to the electronic supplementary material.

Supplementary file1 (DOCX 15 KB)

**Supplementary Fig. 1.** Association of ex-smoking (ex-smokers vs non-smokers) with developing EoCRN risk in case–control studies. The result from the fixed effects model with a sample size of 88914. EoCRN, early-onset colorectal neoplasms; CI, confidence interval; OR, odds ratio (TIF 3600 KB)

**Supplementary Fig. 2.** Association of ex-smoking (ex-smokers vs non-smokers) with developing EoCRN risk in cross-section studies. The result from the fixed effects model with a sample size of 6492. EoCRN, early-onset colorectal neoplasms; CI, confidence interval; OR, odds ratio (TIF 3044 KB)

**Supplementary Fig. 3.** Association of smoking (current smokers vs non-smokers) with developing EoCRN risk in Asia. The result from the fixed effects model with a sample size of 25561. EoCRN, early-onset colorectal neoplasms; CI, confidence interval; OR, odds ratio. (TIF 3600 KB)

**Supplementary Fig. 4.** Association of smoking (current smokers vs non-smokers) with developing EoCRN risk in America and Europe. The result from the fixed effects model with a sample size of 69845. EoCRN, early-onset colorectal neoplasms; CI, confidence interval; OR, odds ratio (TIF 3720 KB)

**Supplementary Fig. 5.** Association of ex-smoking (ex-smokers vs non-smokers) with developing EoCRN risk in Asia. The result from the fixed effects model with a sample size of 25561. EoCRN, early-onset colorectal neoplasms; CI, confidence interval; OR, odds ratio (TIF 2945 KB)

**Supplementary Fig. 6.** Association of ex-smoking (ex-smokers vs non-smokers) with developing EoCRN risk in America and Europe. The result from the fixed effects model with a sample size of 69845. EoCRN, early-onset colorectal neoplasms; CI, confidence interval; OR, odds ratio (TIF 3046 KB)

## Data Availability

All data available upon request.
