# Supplementary material for: Smoking as a risk factor for colorectal neoplasms in young individuals? A systematic meta-analysis
Source: Int J Colorectal Dis. 2023 May 6;38(1):114. doi: 10.1007/s00384-023-04405-w (PMC10163071; doi:10.1007/s00384-023-04405-w)
Supplement: Supplementary file 1 — Supplementary file1 (DOCX 15 KB) [file 384_2023_4405_MOESM1_ESM.docx]

**PubMed**

| Search | Query |
| --- | --- |
| #1 | "young onset"[Title/Abstract] OR "early onset"[Title/Abstract] OR "young adult*"[Title/Abstract] OR "early-onset"[Title/Abstract] OR "young-onset"[Title/Abstract] OR adolescen*[Title/Abstract] OR "under 50"[Title/Abstract] OR "under the age of 50"[Title/Abstract] OR "younger than 50"[Title/Abstract] OR "under 40"[Title/Abstract] OR "younger than 40"[Title/Abstract] OR "under 30"[Title/Abstract] OR "younger than 30"[Title/Abstract] OR "under 20"[Title/Abstract] OR "younger than 20"[Title/Abstract] |
| #2 | "colorectal neoplasms"[MeSH Terms] OR "colorectal neoplasms"[tiab] OR "colorectal cancer"[tiab] OR ("colonic neoplasms"[MeSH Terms] OR "colonic neoplasms"[tiab] OR "colon cancer"[tiab]) OR ("rectal neoplasms"[MeSH Terms] OR "rectal neoplasms"[tiab] OR "rectal cancer"[tiab]) |
| #3 | #1 AND #2 |
| #4 | risk*[Title/Abstract] OR "determinant*"[Title/Abstract] OR "cause*"[Title/Abstract] OR associat*[Title/Abstract] OR relat*[Title/Abstract] OR factor*[Title/Abstract] |
| #5 | #3 AND #4 |
| #6 | ("smok*"[All Fields] OR ("cigarett"[All Fields] OR "cigarette smoking"[All Fields] OR "cigaretts"[All Fields] OR "tobacco products"[MeSH Terms] OR ("tobacco"[All Fields] AND "products"[All Fields]) OR "tobacco products"[All Fields] OR "cigarette"[All Fields] OR "cigarettes"[All Fields])) |
| #7 | #5 AND #6 |

**Embase(OVID)**

| Search | Query |
| --- | --- |
| #1 | "colorectal neoplasms".mp. or exp colorectal tumor/ |
| #2 | "colonic neoplasms".mp. or exp colon tumor/ |
| #3 | "rectal neoplasms".mp. or exp rectum tumor/ |
| #4 | ("colorectal cancer" or ("colonic neoplasms" or "colon cancer") or ("rectal neoplasms" or "rectal cancer")).mp |
| #5 | #1 OR #2 OR #3 OR #4 |
| #6 | ("young onset" or "early onset" or "young adult*" or "early-onset" or "young-onset" or adolescen* or "under 50" or "under the age of 50" or "younger than 50" or "under 40" or "younger than 40" or "under 30" or "younger than 30" or "under 20" or "younger than 20").mp |
| #7 | (risk* or "determinant*" or "cause*" or associat* or relat* or factor*).mp |
| #8 | ("smok*" or "cigarett" or "cigaretts" or "tobacco products" or "cigarette" or "cigarettes").mp |
| #9 | tobacco products.mp. or exp tobacco/ |
| #10 | #8 OR #9 |
| #11 | #5 AND #6 AND #7 AND #10 |

**Web of Science**

| Search | Query |
| --- | --- |
| #1 | ("colorectal neoplasms") OR ("colonic neoplasms") OR ("rectal neoplasms") OR ("colorectal cancer") OR ("colonic neoplasms" OR "colon cancer") OR ("rectal neoplasms" OR "rectal cancer") (All Fields) |
| #2 | ("young onset" OR "early onset" OR "young adult*" OR "early-onset" OR "young-onset" OR adolescen* OR "under 50" OR "under the age of 50" OR "younger than 50" OR "under 40" OR "younger than 40" OR "under 30" OR "younger than 30" OR "under 20" OR "younger than 20") (All Fields) |
| #3 | (risk* OR "determinant*" OR "cause*" OR associat* OR relat* OR factor*) (All Fields) |
| #4 | ("smok*" OR "cigarett" OR "cigaretts" OR "tobacco products" OR "cigarette" OR "cigarettes") (All Fields) |
| #5 | #1 AND #2 AND #3 AND #4 |
