# Supplementary figures and images for: Smoking as a risk factor for colorectal neoplasms in young individuals? A systematic meta-analysis
Source: Int J Colorectal Dis. 2023 May 6;38(1):114. doi: 10.1007/s00384-023-04405-w (PMC10163071; doi:10.1007/s00384-023-04405-w)

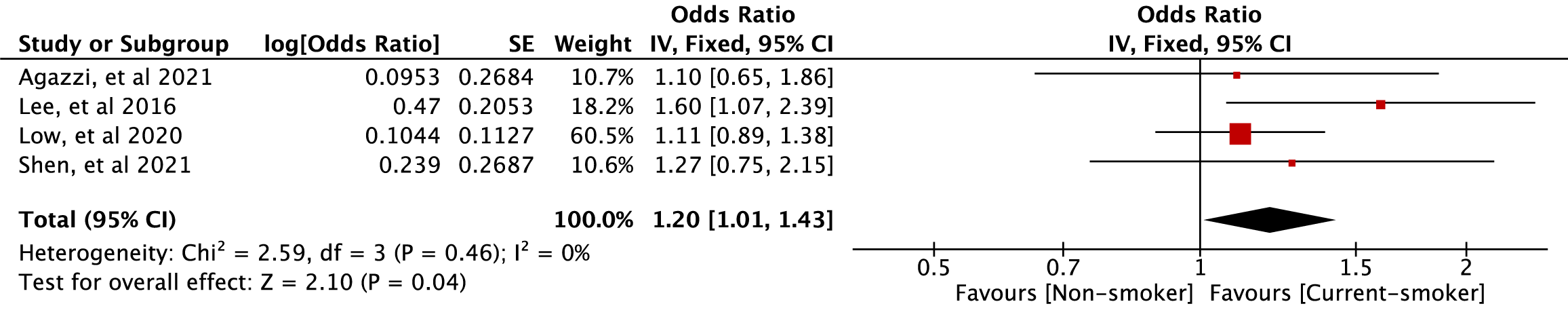

Supplement: Supplementary file 2 — Supplementary Fig. 1. Association of ex-smoking (ex-smokers vs non-smokers) with developing EoCRN risk in case–control studies. The result from the fixed effects model with a sample size of 88914. EoCRN, early-onset colorectal neoplasms; CI, confidence interval; OR, odds ratio (TIF 3600 KB) [file 384_2023_4405_MOESM2_ESM.tif]

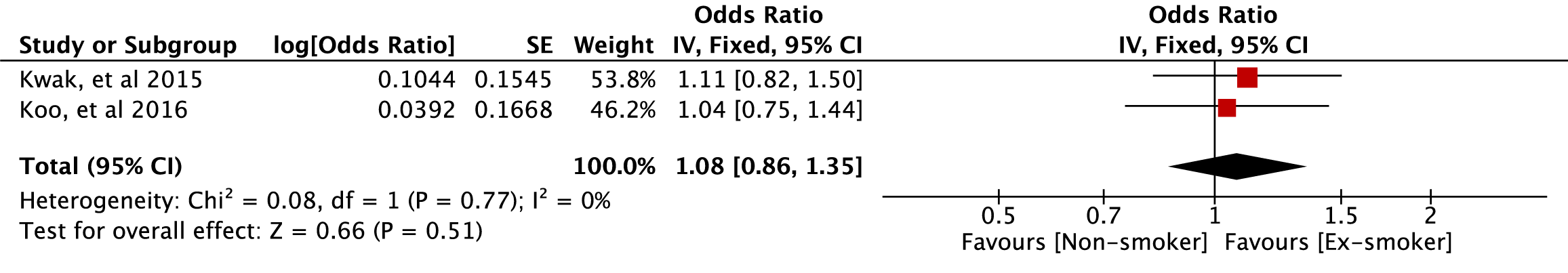

Supplement: Supplementary file 3 — Supplementary Fig. 2. Association of ex-smoking (ex-smokers vs non-smokers) with developing EoCRN risk in cross-section studies. The result from the fixed effects model with a sample size of 6492. EoCRN, early-onset colorectal neoplasms; CI, confidence interval; OR, odds ratio (TIF 3044 KB) [file 384_2023_4405_MOESM3_ESM.tif]

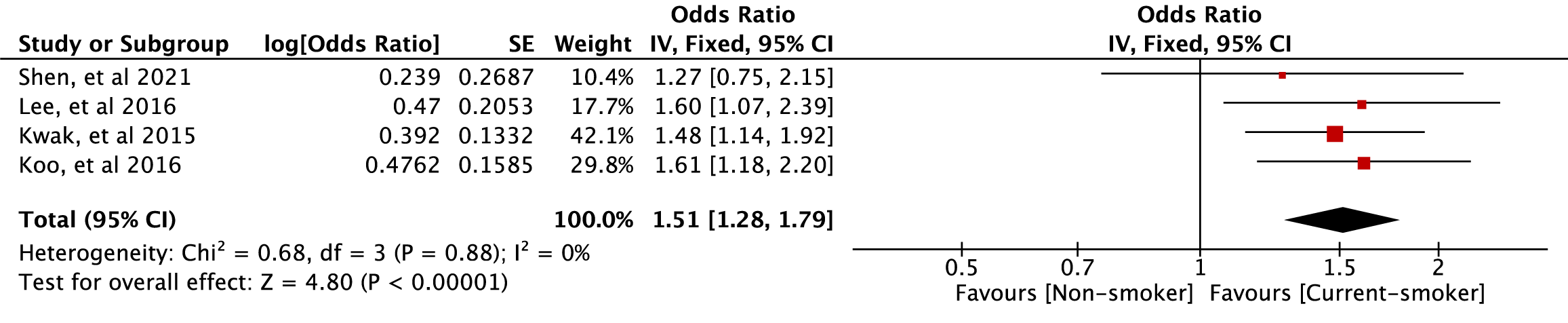

Supplement: Supplementary file 4 — Supplementary Fig. 3. Association of smoking (current smokers vs non-smokers) with developing EoCRN risk in Asia. The result from the fixed effects model with a sample size of 25561. EoCRN, early-onset colorectal neoplasms; CI, confidence interval; OR, odds ratio. (TIF 3600 KB) [file 384_2023_4405_MOESM4_ESM.tif]

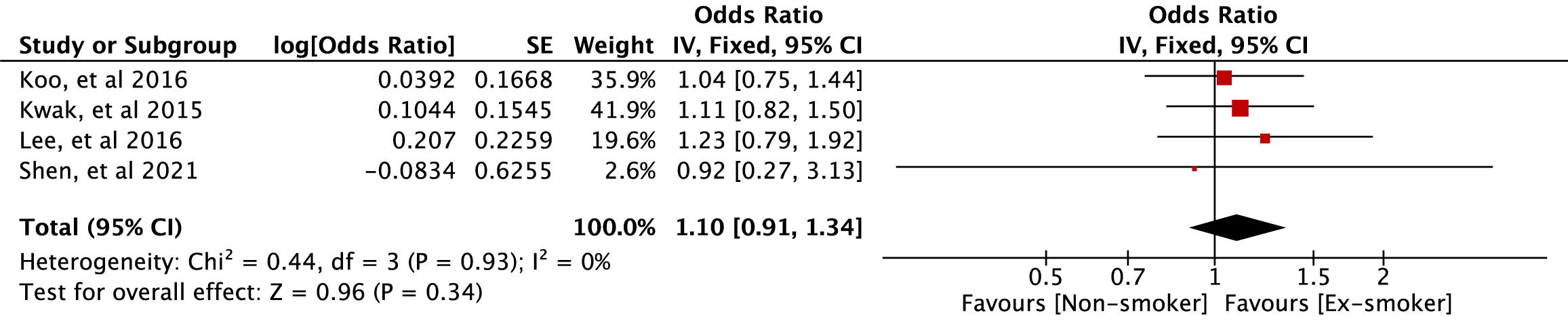

Supplement: Supplementary file 5 — Supplementary Fig. 4. Association of smoking (current smokers vs non-smokers) with developing EoCRN risk in America and Europe. The result from the fixed effects model with a sample size of 69845. EoCRN, early-onset colorectal neoplasms; CI, confidence interval; OR, odds ratio (TIF 3720 KB) [file 384_2023_4405_MOESM5_ESM.tif]

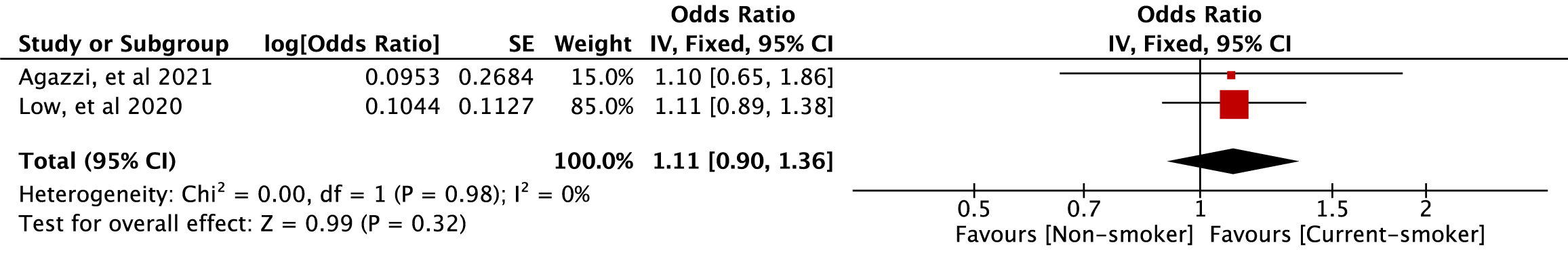

Supplement: Supplementary file 6 — Supplementary Fig. 5. Association of ex-smoking (ex-smokers vs non-smokers) with developing EoCRN risk in Asia. The result from the fixed effects model with a sample size of 25561. EoCRN, early-onset colorectal neoplasms; CI, confidence interval; OR, odds ratio (TIF 2945 KB) [file 384_2023_4405_MOESM6_ESM.tif]

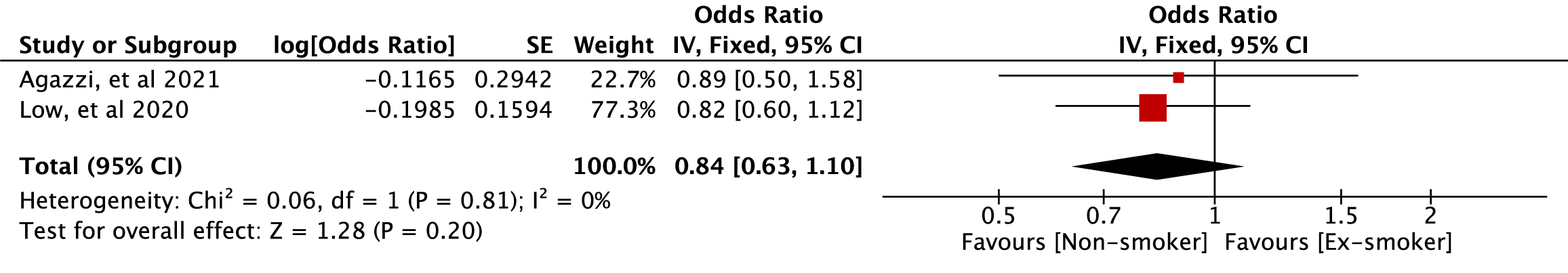

Supplement: Supplementary file 7 — Supplementary Fig. 6. Association of ex-smoking (ex-smokers vs non-smokers) with developing EoCRN risk in America and Europe. The result from the fixed effects model with a sample size of 69845. EoCRN, early-onset colorectal neoplasms; CI, confidence interval; OR, odds ratio (TIF 3046 KB) [file 384_2023_4405_MOESM7_ESM.tif]
